# Mechanisms to exclude local people from forests: Shifting power relations in forest transitions

**DOI:** 10.1007/s13280-021-01613-y

**Published:** 2021-08-23

**Authors:** Melanie Pichler, Martin Schmid, Simone Gingrich

**Affiliations:** Institute of Social Ecology, University of Natural Resources and Life Sciences Vienna, Schottenfeldgasse 29, 1070 Vienna, Austria

**Keywords:** Austria, Lao PDR, Multifunctional landscapes, Political ecology, Shifting cultivation, Theory of access

## Abstract

Forest transitions may significantly contribute to climate change mitigation but also change forest use, affecting the local people benefiting from forests. We analyze forest transitions as contested processes that simplify multifunctional landscapes and alter local livelihoods. Drawing on the Theory of Access, we develop a conceptual framework to investigate practices of multifunctional forest use and the mechanisms that exclude local forest use(r)s during forest transitions in nineteenth century Austria and twenty-first century Lao PDR. Based on historical sources, interviews and secondary literature, we discuss legal, structural and social-metabolic mechanisms to exclude multifunctional forest practices, marginalizing peasants and shifting cultivators. These include, for example, the increasing enforcement of private ownership in forests or the shift from fuelwood to coal in Austria and restrictive land use planning or the expansion of private land concessions in Laos. By integrating political ecology and environmental history in forest transitions research we unravel shifting power relations connected to forest change.

## Introduction

Reforestation can contribute to prevent the most detrimental effects of the climate crisis (Bastin et al. 2019). In the literature, a “forest transition” transcribes the gradual shift from net deforestation to reforestation (Mather 1992; Meyfroidt and Lambin 2011; Gingrich et al. 2019). While global forest areas continue to decline (Köhl et al. 2015), forest transitions have been traced in many countries across the globe, both in terms of forest area expansion (Southworth et al. 2012; Jadin et al. 2016) and forest biomass increase or vegetation thickening (Magerl et al. 2019; Le Noë et al. 2020). Studies identified ideal-type “forest transition pathways”, ranging from state interventions and economic development to globalization and smallholder tree planting that drive forest expansion or recovery (Rudel et al. 2005, 2020; Lambin and Meyfroidt 2010). Substantial contributions also highlighted the importance of economic (Barbier et al. 2010), cultural (Kull 2017), biophysical (Gingrich et al. 2019) and governance (Haider et al. 2018; Riggs et al. 2018) dimensions in explaining and assessing forest transitions. So far, however, politics and changing power relations have received little attention in forest transition research (for exceptions, see Lestrelin et al. 2014; Pichler et al. 2021).

At the same time, yet separated from the forest transition literature, social-ecological research has highlighted the importance of forests for local communities and vice versa (Garnett et al. 2018) and the relevance of local knowledge and institutions (including the recognition of land rights) for conservation efforts (Akamani et al. 2015; Martin et al. 2016; Gadgil et al. 2021). While research suggests that forest loss rates are lower on indigenous peoples’ lands (Fa et al. 2020) or on lands with “relatively secure local rights to use and manage forests” (Sandbrook et al. 2010, p. 331), reforestation and conservation efforts increasingly threaten local peoples’ livelihoods (Barr and Sayer 2012; Fox et al. 2014; Schleicher et al. 2019). These often rely on multifunctional forest landscapes typical for extensive land use practices, such as forest grazing or shifting cultivation (Myllyntaus et al. 2002; Guzmán et al. 2011; Castella et al. 2013; Dressler et al. 2017).

We argue that forest transitions simplify these multifunctional forest landscapes through a separation of agricultural and forest land (Pichler et al. 2021).^1^ We approach this separation along two dimensions: First, the separation of agricultural and forest land simplifies the *uses* of the forest, from multiple forest use practices to usually one dominant, for example, timber production or carbon sequestration. Second, this separation also simplifies the group of *users*, from multiple local forest users to state and/or private control (Barr and Sayer 2012; see also Scott 1998). While social-ecological simplification may support forest recovery, it comes at the expense of local forest users and their livelihood practices (Castella et al. 2013; Pichler et al. 2021). Separating local people from forests in the course of forest transitions has thus been a contested process, driven by “powers of exclusion” (Hall et al. 2011).

In this article, we build on political ecology to develop a conceptual framework for analyzing the powers of exclusion during forest transitions and apply it in two case studies. Our framework draws on Ribot and Peluso’s (2003) Theory of Access, a concept for the analysis of the “multiple mechanisms by which individuals, groups, or institutions gain, control, or maintain access within particular political and cultural circumstances” (p. 161). As the inverse of access, we describe the mechanisms that *exclude* local people from benefiting from forests (Hall et al. 2011; Myers and Hansen 2020, pp. 152–153). Whereas legal exclusion constitutes the most immediate purposeful form of exclusion, structural and relational mechanisms stabilize this exclusion, for example, by reducing access to capital, markets or technology (Ribot and Peluso 2003). From a social-ecological perspective, these exclusionary mechanisms link to more fundamental shifts in societal resource and energy use during forest transitions that include processes of agricultural intensification and commercialization as well as an increasing use of fossil energy (Gingrich et al. 2019; Pichler et al. 2021).

We analyze the practices of multifunctional forest use and their benefits for local livelihoods as well as the mechanisms that reduce these practices and exclude local people from forests in two case studies that have experienced forest transitions in different time periods and geographical locations: nineteenth century Austria and twenty-first century Lao PDR. Although these case studies obviously differ in terms of socio-cultural, political, economic and ecological contexts, we show that forest transition processes result in common trajectories of simplifying multifunctional forest landscapes. Without missing historical and geographical peculiarities, we therefore discuss local *practices* of multifunctional forest use and investigate the *mechanisms* that exclude local people and their practices from the forests. Local people refer to people that rely on and benefit from forests for their livelihoods, often complementing other sources of income. These especially refer to peasants in nineteenth century Austria who practiced mixed farming (combining cropping and livestock rearing) and shifting cultivators in contemporary Lao PDR.

The article is structured as follows: The next section introduces the Theory of Access and develops a conceptual framework to analyze shifting power relations in forest transitions. “Materials and methods” section introduces the case studies and describes materials and methods. “Results” section analyzes the practices of multifunctional forest use and the mechanisms that exclude local people from forests, first in nineteenth century Austria and then in contemporary Lao PDR. “Discussion” section draws comparative conclusions in discussing exacerbating powers of exclusion in forest transitions and highlighting the importance of forests as nutrient and energy reservoirs for subsistence livelihoods. In doing so, we contribute to the interdisciplinary advancement of forest transition research through integrating key insights from political ecology and environmental history to address shifting power relations in forest transitions. At the same time, the research illustrates that the separation of local people from multifunctional forests may also compromise some of the proclaimed effects and benefits of reforestation and conservation efforts.

## Conceptual framework: Shifting power relations in forest transitions

**Fig. 1:**
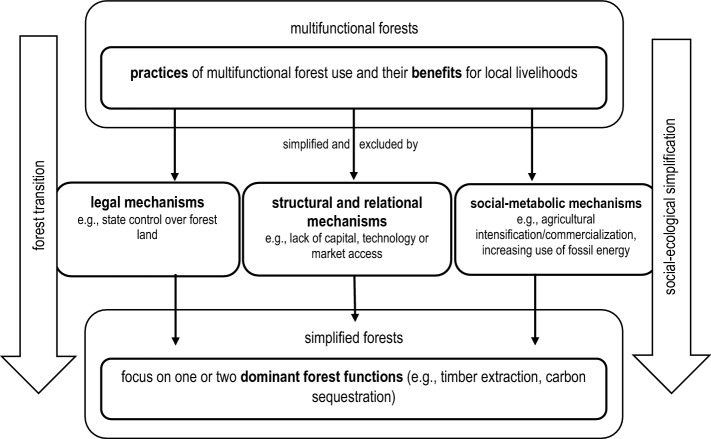
Shifting power relations in forest transitions. Mechanisms to exclude local people from forests. Adapted from the Theory of Access by Ribot and Peluso (2003). Own illustration

Advancing interdisciplinary forest transition research, we develop a conceptual framework to show shifting power relations in forest transitions. Figure 1 conceptualizes forest transitions as processes of social-ecological simplification where local people that benefit from multifunctional forests for their livelihoods face mechanisms that aim at simplifying forests and exclude them from these practices and benefits.

We draw on the Theory of Access (Ribot and Peluso 2003), a concept from political ecology that has been widely applied since it was first introduced in 2003 (Corbera and Brown 2010; Maryudi and Krott 2012; De Vos 2016). Ribot and Peluso (2003) define access as “the *ability* to benefit from things” (p. 153). For the empirical analysis, they propose to *first*, “identifying the object of inquiry—a particular benefit coming from a particular resource” and *second*, to analyze the “multiple mechanisms by which individuals, groups, or institutions gain, control, or maintain access within particular political and cultural circumstances” (p. 161). We approach the “particular benefit” through describing *practices* of multifunctional forest use and the *benefits* that local people gain from these practices. We identify practices of multifunctional forest use that are documented during periods of forest transitions. Focusing on the most contested practices, that is, those most addressed in the empirical evidence we work with, we limit our assessment to those multifunctional forest uses connected to the *material* use of forest resources (i.e., to “provisioning ecosystem services” provided by forests (Millennium Ecosystem Assessment 2005)) while excluding other, non-material forest uses such as spiritual or recreational uses.^2^ The focus on the material use of forests enables us to broadly discuss in which way these multifunctional practices contribute to the livelihoods of local people (e.g., enabling cash income or adding to subsistence).

As processes of social-ecological simplification, we investigate forest transitions as processes in which multifunctional forests are simplified and local forest users are excluded from benefits through specific *mechanisms of exclusion*. As the inverse of access, we describe the mechanisms that exclude local people and their practices from the forests and therefore from the ability to benefit from forests (see also Myers and Hansen 2020, pp. 152–153). These mechanisms of exclusion closely relate to what Hall et al. (2011) identify as “powers of exclusion”. We understand power as “the capacity of some actors to affect the practices and ideas of others” (Ribot and Peluso 2003, p. 155). Mechanisms of exclusion, more specifically, focus on *processes* and *actors* that are able to set these processes in motion (Hall et al. 2011, p. 5). These actors are more apparent for some of these processes (e.g., state authorities for formalized legal exclusion) than for others (e.g., market mechanisms or technical improvements).

*Legal mechanisms* are the most common form of rights-based exclusion (Pichler 2015; Ribot and Peluso 2003). With regard to forest resources and use, legal exclusion is most common through state control over forest land (Peluso and Vandergeest 2001). Most often, state control over forest land does not completely exclude local people’s ability to benefit from forests but exacerbates their ability to access through a complex web of property and tenure relations that alters power relations in favor of corporate, state or military interests. Besides legal mechanisms, *structural and relational mechanisms* of exclusion influence people’s ability to benefit from forest resources. “Different political-economic circumstances change the terms of access and may therefore change the specific individuals or groups most able to benefit from a set of resources” (Ribot and Peluso 2003, p. 158). Changing economic benefits from forest management (e.g., through timber extraction or carbon sequestration) enable some people to benefit from environmental transformations while excluding others (Barr and Sayer 2012; Oosthoek and Hölzl 2018). Structural and relational mechanisms of exclusion include, for example, the lack of technology (including infrastructure like roads, equipment, seedlings or machinery), lack of capital (which is closely related to technology, but also includes the purchasing of access rights such as concessions), the lack of market access, labor opportunities, and the exclusion from (or non-recognition of local) knowledge (Ribot and Peluso 2003, pp. 161–172).

While Ribot and Peluso (2003)—as well as ensuing research—have focused on legal as well as structural and relational mechanisms that prevent people from benefiting from resources, we adopt a social-ecological perspective, connecting the separation of local people from forests to changes in societal resource and energy use. Studies show that, historically, forests have recovered with the intensification of agriculture (Rudel et al. 2009) and the energetic shift from biomass (e.g., fuelwood) to fossil energy (e.g., coal) (Erb et al. 2008; Myllyntaus et al. 2002). The social-ecological simplification of multifunctional forests has thus been supported, or enabled, by *social-metabolic mechanisms* (Gingrich et al. 2019) such as the intensification of agriculture or the increasing use of fossil energy. While traditional land-use practices rely on forests or other commonly-used land areas for the provision of nutrients for agriculture, industrializing land-use practices introduce mineral or other fertilizers and thus reduce the biophysical links between agricultural and forest lands (Guzmán et al. 2011).

This conceptual framework helps us to explain similarities in simplification and exclusion processes, that is, shifting power relations, during forest transition processes without missing historical and geographical peculiarities.

## Materials and methods

We adopt a case study approach to analyze the practices of multifunctional forest use and their benefits for local livelihoods as well as the mechanisms that exclude local people from forests in two countries that have experienced forest transitions in different time periods and geographical locations: nineteenth century Austria and twenty-first century Lao PDR. Apart from the different time periods in which these countries have experienced forest regrowth, the selection of the case studies also covers different ecological characteristics (temperate versus tropical forests and land-use practices) and forest paradigms (optimizing timber production versus combining timber production and carbon sequestration). At the same time, the two countries, however, also share important similarities: Both Austria and Lao PDR are land-locked and mountainous countries with fairly high forest cover, low population density and economic integration at the onset of the forest transition. Whereas, however, previous forest transition research has already focused on socio-economic conditions of forest transitions (e.g., forest recovery coinciding with economic development and globalization, Wolfersberger et al. 2015; Li et al. 2017), political dimensions and changing power relations have received limited attention. This general observation has led us to analyze common features of social-ecological simplification and exclusionary mechanisms during forest transitions as “historical configuration[s] of multiple and complex trajectories” rather than isolated cases (Peluso and Vandergeest 2020, p. 1086), without missing the historical and geographical peculiarities of the two case studies.

To analyze practices of multifunctional forest use and the mechanisms that separate and exclude local people from forests, we examine (forest) laws and regulations, secondary and grey literature. Additionally, we rely on historical sources for the Austrian case study and on qualitative expert interviews for the Lao case study. We conducted 22 interviews during two research stays in November 2019 and in February and March 2020 with representatives of state institutions, international organizations, companies, NGOs, and donor organizations in Laos. Most of the interviews were conducted in English but we had collaboration partners and research assistants that helped with interview appointments, institutional contexts and translation.

In the analysis of both the Austrian and the Lao case study, we focus on those practices and mechanisms that (a) policy makers and forestry experts emphasize for their importance in minimizing forest degradation and that (b) play an important role for local livelihoods. In doing so, we do not imply that these exclusionary mechanisms always succeed, as “there are [often] finer channels through which communities exert a measure of control over social-ecological space” (Ingalls 2017, p. 11). We are, however, interested in the mechanisms that *exacerbate* the separation of local people from forests that has important justice implications for forest transitions (Scheidel and Gingrich 2020).

## Results

### Reducing practices of multifunctional forest use and excluding peasants from forests in nineteenth century Austria

For the territory of present-day Austria, quantitative assessments based on archival documents and statistical publications identify a gradual increase in both forest area and forest biomass density between 1830 and the early twentieth century (Gingrich et al. 2007). Like in many other European countries, the forest transition in Austria was accompanied by substantial political and institutional change. A land reform in 1848 (*Grundentlastung*) abolished serfdom, one of the cornerstones of the feudal regime, eliminating the tithes and robot services peasants had owed landlords (Jepsen et al. 2015). In principle, peasants were able to buy the land they had formerly worked in their landlord’s attendance. In practice, the right to buy the land was limited, and high costs to clear off feudal obligations resulted in high debts (Sandgruber 1995) that denied the peasants a true land reform for many decades. The land reform in 1848 was restricted to agricultural land and excluded forests. Long and controversial debates went on to establish a new forest regime in the decades after 1848, aiming at maximizing timber production while repressing other forest uses. A milestone in this debate was the Forest Act (*Forstgesetz*), implemented in 1852, which aimed at regulating forest use after the land reform.

In contrast to other regions of Central Europe, most Austrian forests were private property, and a good share of these forests was owned by peasants themselves, either individually or collectively (*Bauernwald*) (Weigl 2002, p. 597). In these peasant forests, it was particularly difficult to enforce a new, ‘rational’ and modern regime. In 1895, almost half of all forests in Lower and Upper Austria were peasant-owned, a little less was owned by former landlords, the church or other large holders, and only around 10% were owned by the state. Land tenure was similar in the alpine, mountainous regions but with a higher share of peasant *communal* forest (*Gemeindewald*) because of the high importance of livestock breeding (Weigl 2002, p. 618, cited from Guttenberg 1899). However, even if peasants often were the legitimate owners of forest areas, the political debates around forests, their conservation and the mechanisms of exclusion targeted peasant practices across all provinces. Peasants and their practices were considered responsible for forest degradation and for jeopardizing a modern forests regime.

#### Practices of multifunctional forest use

In nineteenth century Austria, besides the “main” forest use of stem wood extraction, the Forest Act of 1852 regulated a large number of “side uses” to supply markets and local communities with a variety of resources (Gingrich et al. 2020). Side uses of forests were practiced by multiple users and regulated by servitudes (*Servitute*), determining who had the right to use a specific area of forest at which level of intensity (e.g., the time period or maximum duration of use or the amount of resources extracted). The most important side uses were the collection of deadwood and harvest residues as fuelwood as well as forest grazing and the extraction of leaves, litter or grass for fodder or bedding material (Schopf 1853; Mally 1854).

These practices of multifunctional forest use provided major benefits for local peasant livelihoods. Fuelwood was the major energy source in rural households, and collecting it in nearby forests freed peasants from economic burdens. Forest grazing and actively harvesting fodder and bedding material benefited those peasant households with livestock. In fact, a “shortage of straw” is named as a reason for litter extraction in the prealpine village Konradsheim in Lower Austria in the Franciscan Cadastre, a detailed land survey conducted throughout the Habsburg Monarchy (NÖLA 1829, pp. 35–36). Reconstructions of nutrient fluxes based on this source further highlight the relevance of livestock-related forest use. In addition to sustaining the livestock system, these practices also improved agricultural productivity: By feeding the livestock and providing bedding material, biomass from forests ultimately resulted in additional manure, which was used to fertilize croplands (Krausmann 2004; Gingrich et al. 2015). Beyond collecting fuelwood and forest grazing, a number of other side uses are documented in legal regulations (Mally 1854). Some pertained to using living trees to extract kindling, resin or sap, peeling off bark or collect wild fruit. Other forest products included seeds, moss, herbaceous plants, berries and mushrooms, but also loam, clay and rocks.

#### Mechanisms to exclude peasants from multifunctional forests

The Forest Act of 1852 codified mechanisms that increasingly excluded peasants and their practices of multifunctional forest use from these forests. The process of enforcing and revising this Act continued for decades, well into the twentieth century (Feichter 1995). The printed (i.e., edited) minutes of the Agricultural Congress held for several days in Vienna in March 1849 (Verhandlungen des landwirthschaftlichen Congresses gehalten zu Wien im Monate März 1849) inform about debates on the draft for this new Act between the minister, his officials and about twenty selected representatives of specific interest groups. Members of this Congress came from almost all parts of the Austrian empire and included foresters, senior forest officials, members of agricultural associations, industrialists and representatives of major noble or other forest owners such as monasteries. The largest and most diverse group of users, peasants who used forests they did not own (so-called *Eingeforstete*), had no voice. Their practices were particularly contested and liberal landowners aimed to permanently exclude them in exchange for financial compensation.

The main legal mechanism of exclusion was the implementation of private property rights on forests that aimed at securing the primacy of timber production for markets. The new Forest Act stipulated at the very beginning that “no woodland may be taken away from timber production or used for other purposes without a permit” (§2), continuing that “no forest may be devastated, i.e., treated in such a way that the further cultivation of timber is endangered or made completely impossible” (§4). To achieve these goals, the Act’s preamble mentioned the “special protection of private property” very prominently as the first motive, even before the “conservation and care of forests” itself. In their contributions to the 1849 debate, the majority of the members of the Agricultural Congress repeatedly referred to the protection of private property as supreme “principle”. A statement by Count Kazimierz Krasicki, deputy for Gallicia at the Congress, agronomist and one of the pioneers of the abolition of serfdom in his homelands, exemplified the goal of simplifying multifunctional forest landscapes and excluding peasants from these forests:

There are indeed major obstacles which do not allow good management of forests. These are the ‘servitudes’. They do not exist to the same extent in all provinces [...]. But they exist, whether we like it or not. I believe that when the forest owner, after abandonment of all servitudes, becomes master of his own house, he will certainly manage the forest economy well. In the present situation, however, the forest owner does not manage the forest for himself, but for those entitled to the servitudes. If this obstacle is removed, the influence of the government can be reduced to the absolutely necessary. (Verhandlungen 1849, 287)

Shortly after a bourgeois revolution, the question of whether and to what extent the state was allowed to intervene in private property was by far the most important question and constituted the most explicit legal mechanism to exclude peasants and their practices of multifunctional forest use from privately owned forests. The Ministry of Land Improvement and Mining, that was in charge of forestry, shared this liberal ideal, but also upheld the “common good” that justified the intervention in private property rights and thus state control of forests. With reference to the common good, however, the ministry did not support the interests of local forest users, but rather the “people in need of timber, the vast majority of the population, timber consumers, urban dwellers and industrialists” (Verhandlungen 1849, esp. 288–292).

The long-term effort to exclude side use(r)s from forests through legal mechanisms was complemented by social-ecological mechanisms, that is, technical changes in agricultural management, that gradually loosened agricultural dependence on forests and supported the separation of agricultural and forest land. To reduce extraction of litter from forests, the Ministry of Agriculture recommended technological improvements in manure and livestock management, including the construction of stable floors requiring less bedding material, changes in fertilizer management (liquid slurry rather than solid manure), and the application of mineral fertilizer such as marl instead of solid manure (Trientl 1873). In hindsight, the increasing cultivation of leguminous crops such as clover on agricultural fallows, capable of binding aerial nitrogen in the soil, represents another adaptation that reduced the reliance on nutrient transfers from forests (Krausmann 2006). These technical improvements and the abandonment of the servitudes were, however, slow processes as they were also connected to high investments. Abrupt changes would have destroyed the livelihoods of many peasant families who needed the forest as a reservoir of energy and nutrients. This must have been obvious even to the most outspoken followers of modern, rational and ‘sustainable’ forestry.

As another social-ecological mechanism of exclusion, the substitution of fuelwood by fossil fuels contributed to simplify the multifunctionality of forests (Gingrich et al. 2019). In the mid-nineteenth century, the energy transition was still in an early phase in Austria. However, owners of large forests regarded such a ‘fossilization’ of heat provision as a future fact and used it in their negotiations with the Ministry of Land Improvement and Mining on the new Forest Act. The ministry argued that some form of state control over private forests was unavoidable because of increasing fuelwood demand by growing industries and cities—fuelwood collection by local peasants was not even mentioned in this context. Forest owners and managers in turn put forward that “one must not forget that we also have fuel other than wood, as far as we speak of firewood; there is a mass of coal that will replace firewood” (Verhandlungen 1849, p. 280). Similarly, Carl Ritter von Baretta, a pronounced liberal, reminded his colleagues about the empire’s “immense seams of coal”. “If only wood prices were higher”, he complained, “all these enormous coal seams would be exploited”, bringing an end to the repeatedly proclaimed wood shortages (Verhandlungen 1849, S. 282). Decades before coal actually replaced fuelwood, the incipient energy transition already had its role in separating—and eventually excluding—peasants from forests. Powerful actors, like the mentioned liberal Baretta, ministry officials and representatives of large forest owners, expected the new energy from fossil fuels would free the forest from side uses other than private timber production.

### Reducing practices of multifunctional forest use and excluding shifting cultivators from forests in contemporary Lao PDR

In contrast to the historical forest transition in nineteenth century Central Europe, Laos provides an example for contemporary forest regrowth in Southeast Asia (Pichler et al. 2021). According to international FAO data, Laos has seen net increases in forest cover since 2000 (Köhl et al. 2015), whereas national inventories remain more pessimistic (Bauernschuster et al.).^3^ In any case, the Government of Lao PDR (GoL) has tightened control over the vast forest resources and has committed to forest expansion since the 1990s, setting an ambitious target of 70% forest cover by 2020 (MAF 2005). Since the 2000s, Laos has, at the same time, seen an unprecedented rush in large-scale land acquisitions, mainly for mining, commercial agriculture and tree plantations, that increase pressure on forests and local livelihoods (Ingalls et al. 2018).

Comprehensive land use planning and forest policies have supported these transformations. From the 1990s onwards, the Land and Forest Allocation (LFA) program started to assign plots of both agricultural and (village) forest land to individual households (MAF 2005, p. 5). The LFA program aimed at separating agricultural and forest land, thus, directly targeting practices of multifunctional forest use. It resulted in 70% of the country being classified as *state forestland*, where agricultural practices are excluded or severely restricted (MRLG 2019).

Despite high economic growth rates of 7.7% per year over the last decade, the majority of the Lao population (almost 80%) are still engaged in agriculture, both for subsistence or for generating cash income. In rural households, even 90% engage in some form of agricultural production (Ingalls et al. 2018, pp. 88–91). The separation of agricultural and forest lands and the subsequent increase in commercial agriculture and state control over forests have especially affected shifting cultivators in the Lao uplands that rely on forests and fallows for their livelihoods (Castella et al. 2013).

#### Practices of multifunctional land use

Shifting cultivation (also known as swidden agriculture or slash-and-burn cultivation) forms the basis of multifunctional land-use practices in the Lao uplands. Shifting cultivators usually burn small plots of land for upland rice production. In the absence of manure or chemical fertilizer, ash from above-ground biomass provides nutrients for agricultural production. After harvesting, the plot is left idle for usually seven to twelve years to recover. The limited amount of time that land is used for agricultural cultivation serves as an important means for pest and weed control. Despite the drastic land-use changes in recent years, shifting cultivation still supports 240 000 households, especially in the northern uplands and “conservative estimates suggest that the total amount of land involved in shifting cultivation in Lao PDR may be around 7 million hectares” or almost 29% of the total area (Ingalls et al. 2018, p. 100). Beyond rice production, the extensive fallow systems provide local people with multiple additional benefits to support their livelihoods, including grazing of livestock in harvested areas and diverse forest products (Castella et al. 2013, p. 72; Vongkhamho et al. 2019). These Non-Timber-Forest Products (NTFP) include, for example, cardamom, bamboo, benzoin, mushrooms, berries or teas, and often provide an important source of cash income for rural communities. A survey from The Agro-Biodiversity Initiative in the Lao PDR (TABI) has shown that 48% of income generated from the sale of NTFP derive from shifting cultivation systems, compared to only 10% from mature forests (Ingalls and Roth 2018). At the same time, forests and fallows provide for fuelwood. Although the share of biomass (i.e., fuelwood) in total energy consumption decreased from 78% in 2000 to 46% in 2015, biomass is still the major energy source consumed in Laos (Ministry of Energy and Mines, Lao PDR 2018, pp. 74, 81–82).

#### Mechanisms to exclude shifting cultivators from multifunctional forests

Rooted in French colonial forestry, policies aiming at both the increase in national forest cover and the introduction of more productive forms of agriculture have targeted practices of multifunctional forest use in Laos since the 1990s (Castella et al. 2013). Resettlement programs and the Land and Forest Allocation (LFA) program have been the most important legal mechanisms to impede shifting cultivation practices and exclude local people from forests (MAF 2005, p. 40). Over the course of the 1990s, the GoL together with international donor organizations resettled ten thousands of people, mainly from the forested uplands to the lowlands (Baird and Shoemaker 2007; Castella et al. 2013, p. 68; Chazée 2017).^4^ At the same time, the LFA program established the most comprehensive legal mechanism of simplifying multifunctional forest landscapes and excluding shifting cultivators from these forests (Vandergeest 2003, p. 50). LFA was a comprehensive land use planning system that allocated agricultural and village forest land to households and villages, providing them with three-year temporary land use certificates.^5^ Households were granted a maximum of three plots of land, reducing the possible fallow length to two years (Fujita and Phanvilay 2008, p. 121; Kenney-Lazar 2013). The separation of forest and agricultural lands in the course of the LFA program therefore resulted in a distinction between shifting cultivation (*hay kheuan nhai*) and rotational cultivation (*hay moun vien*) (MAF 2005, p. 39). Shifting cultivation relies on extensive multifunctional landscapes (including forests of different stages) which the LFA program and the Forest Law officially prohibit (National Assembly 2007). Rotational cultivation, in turn, is tolerated on plots classified as agricultural land. The shortening of the fallow length by law, however, makes the practice unsustainable as neither agricultural land can restore nor fallows can provide services such as NTFP or carbon sequestration.

The restrictive land use planning and tighter state control over forests therefore also severely restrict local people’s access to NTFP, both in old-growth forests and in fallows. In general, the Forest Law permits the collection of NTFP in village and production forests, provided that regulations and management plans are followed (MAF 2005, p. 20). For example, collectors have to pay a fee of 5% for cardamom or 3% for broom grass to district authorities that is subjected to the preservation of natural resources and replanting (Castella et al. 2013, p. 72). The collection of NTFP is only strictly prohibited in protection forests whereas non-commercial collection is possible in conservation forests (National Assembly 2007). Whereas these legal mechanisms result in a *rearrangement* rather than a strict *exclusion* of local people benefiting from forest resources, the Lao government itself admits exclusionary effects of the LFA program:

In many cases, however, over-zealous zoning of ‘protected’ categories at the expense of village used forests and farming areas has partly precluded collection of fuelwood and NTFPs. Additionally, allotment of land for forest regeneration reduces land available for designation as cropland and could eventually encourage villagers to exploit other areas of forest for cropping. It could also restrict access to such forests for fuelwood or NTFP collection. (MAF, 2005, p. 41)

In line with the LFA program, the ‘remaining’ land, that is, 70% of the Lao territory, was classified as state forestland, differentiating between conservation, protection and production forestland (MRLG 2019). Spanning these three basic forest categories, the Forest Law introduced degraded forestland, regeneration forestland, dry forestland and village forestland as additional classifications of state forestland (National Assembly 2007, p. Art. 56). Especially degraded and regeneration forests mark former shifting cultivation fallows at different stages that the state aims to protect for natural regeneration or convert into tree plantations to reach the 70% forest cover (National Assembly 2007, p. Section 3).

Despite these legal mechanisms of separation and exclusion through LFA and state control over forestland, local people often still find informal ways to access forests and multifunctional landscapes. In the course of the 1990s, the resettlement and LFA policies, however, also prepared the legal ground for more structural and relational mechanisms of exclusion in the form of an unprecedented rush in large-scale land acquisitions that materialized in the 2000s. From the mid-2000s, the policy vision of Turning Land Into Capital (TLIC) encouraged foreign direct investments in concession agriculture and tree plantations to integrate Laos into the global economy through international trade and therefore increase government revenues from these land-based resources (Barney 2009; Kenney-Lazar et al. 2018; Dwyer and Vongvisouk 2019). These investments were driven by the unprecedented structural reconfigurations of neighboring economies and their rapidly increasing need for natural resources to fuel their economic growth model.

In 2017, more than 1 million ha of land was under concessions, contracted for commercial agriculture and forestry, mining and hydropower (Ingalls et al. 2018, pp. 104–105). In the course of these developments, the multifunctional forests have also been increasingly replaced with concessions for tree plantations. Since 2018, the GoL has actively promoted private tree plantations on 600 000 hectares of state forestland that had been classified as degraded forests. Tree plantations mainly consist of fast-growing trees such as eucalyptus and acacia and should combine the more productive use of forestland with the increase in forest cover. As described above, concessions for tree plantations represent a structural mechanism of excluding local people from forests. At the same time, exclusion through concessions is connected to relational mechanisms of (unequal) access to capital, markets and technology. A representative from a private tree plantation company investing in eucalyptus plantations, for example, states:

We know how to manage the trees, so what we like to do is lease the land over 30+ years [...] and have those families or villagers work in plantations, getting skills, getting money on land that would not necessarily be used. (personal communication, Vientiane, February 2020)

Thus, as private large-scale land concessions (both for commercial agriculture and tree plantations) expand into multifunctional forest landscapes, forest functions are simplified towards timber extraction. Whereas local people are indeed encouraged to plant trees and invest in forest restoration, the above quote shows that the lack of capital, technology and targeted knowledge makes their exclusion and re-integration as laborers into tree plantations the most likely outcome.

Additionally, REDD+ plays an increasingly important role in simplifying forests towards carbon sequestration and eventually excluding practices of multifunctional forest use. For more than a decade, Laos has established REDD+ readiness activities in six northern provinces that are expected to provide results-based payments from 2020 onwards (personal communication, Vientiane, November 2019). As a representative of the Department of Forestry explains:

In the northern part, you find a lot of shifting cultivation, whereas in the southern part you find a lot of logging [...]. In the northern part, we are now testing, we focus on it because shifting cultivation is the main [driver of deforestation], and so we are testing in terms of promoting smart agriculture or giving better livelihood [opportunities] and also creating a sustainable forest management. (personal communication, Vientiane, November 2019)

The selection of the six northern provinces is no coincidence as these represent major shifting cultivation areas. Despite other major drivers of deforestation, especially logging and land conversion for agricultural and industrial activities (Lestrelin et al. 2013), REDD+ activities tend to explicitly target forests used for shifting cultivation, further separating and excluding local people from forests.

## Discussion

Although different in time and space, the analysis of the nineteenth century forest transition in Austria and the twenty-first century forest transition in Laos shows that practices of multifunctional forest use have been widespread before and during forest transitions, and that reforestation has been accompanied by social-ecological simplification and the continuing exclusion of local people from forests. Table 1 summarizes the practices of multifunctional forest use and the mechanisms of exclusion. Due to different political systems, the protection of private forest ownership to increase profits from timber extraction at the expense of local forest uses was an outspoken goal in the Austrian case, where the forest transition succeeded a bourgeois revolution. In the one-party state of Lao PDR, in turn, the state took increasing control over the vast forest areas to separate forests from people and increase productivity in both forestry and agriculture. The increasing importance of private tree plantations as well as international climate governance through REDD+ may, however, lead to a more fragmented control over forests (Dwyer et al. 2016; Cole et al. 2017).

**Table 1:** Practices of multifunctional forest use and mechanisms of excluding these in the course of the forest transition in nineteenth century Austria and twenty-first century Laos

	Nineteenth century forest transition in AUSTRIA	Twenty-first century forest transition in LAO PDR
Local people (peasants and shifting cultivators) use multifunctional forests to support their livelihoods	Fuelwood collection, forest grazing, harvest of leaves, litter or grass for fodder or bedding material, collection of multiple other forest products	Fuelwood collection, shifting cultivation, collection of non-timber forest products
The state aims at simplifying forests through exclusive use rights	Secure timber production and supply growing industries and cities (*Nationalökonomie*) with wood and fuel	Secure timber production for international markets and enable carbon sequestration
Legal mechanisms exclude local forest use(r)s	Increasing protection of private property control over forests through the Forest Act of 1852 and subsequent restrictions in the Act	Separating agricultural and forest use through LFA and resettlement programs in the 1990s
Structural, relational and social-metabolic mechanisms support the exclusion of local forest use(r)s	Technological improvements in manure, soil and livestock management, substitution of fuelwood by coal	Expansion of large-scale land concessions (including tree plantations)

Interestingly, the effects are quite similar in both private and state-led control over the forests in that they exclude certain local people (peasants and shifting cultivators) and their practices from or at least reduce community control over the forests. Both in Austria and Laos, those actors that have pushed for exclusive access to forests and aim to decrease multifunctional forest use are powerful (see also, Gerber 2011; Barr and Sayer 2012). That is, they participate in political decisions about future forest and land policies, they have access to capital, technology, institutions and (scientific) knowledge to benefit from the increasingly simplified forests. In Austria, these powerful actors drew on narratives from the Enlightenment discourse of the eighteenth century which presented peasants and their practices as backwards, inefficient and destructive to forests. In Laos, as elsewhere in Southeast Asia (Fox et al. 2009; Dressler et al. 2017), the exclusion of shifting cultivation perpetuates discourses and policies that have been discriminating ethnic minorities over decades in the course of colonial and postcolonial state formation (Lestrelin 2010). Multifunctional forest use(r)s are marginalized as forests are needed for other—economically more viable—purposes. Our findings on the consolidation of corporate or state control at the expense of local use(r)s thus have important implications for contemporary reforestation and conservation efforts (e.g., REDD+) that tend to exacerbate this trend of centralized control over forests (Phelps et al. 2010; Sandbrook et al. 2010; Fox et al. 2014; Scheidel and Work 2018). In doing so, these efforts also run the risk of reversing some of the trends towards decentralization of forest governance and community forestry since the 1990s, especially in the tropics (Poffenberger 2006; Phelps et al. 2010; Sandbrook et al. 2010).

In addition to the social-economic and political dimensions, the marginalization and eventual exclusion of multifunctional forest use also diminishes the role of forests as nutrient and energy reservoirs for local livelihoods, that is, the social-metabolic dimension of exclusion. Both in Austria and Laos, the forests have provided important energy (especially fuelwood) and nutrient flows (for animal husbandry or for rice cultivation). The expulsion of multifunctional forest use therefore comes along with shifts in resource and energy use. While both countries experienced a shift towards more fossil energy during the forest transition, the structural effect of changing energy use was more immediate in Austria than in Laos. Agricultural intensification through large-scale land concessions that transform shifting cultivation areas into plantations in Laos, on the other hand, drives the structural separation of agricultural and forest land at a much faster pace than in nineteenth century Austria where technical improvements in agricultural management added to the separation of peasants from forests over a much longer period of time (Krausmann 2006; Castella et al. 2013). These findings have important implications for a more systemic reading of the social and ecological costs and benefits of forest transitions (Gingrich et al. 2019). Industrial agriculture is a key driver for climate change and often conflicts with reforestation and forest conservation efforts. “Land sharing” in the form of multifunctional forest and agricultural use might therefore increase both local livelihood and climate benefits (Mbow et al. 2014; Dressler et al. 2016).

## Conclusion

Based on two case studies of forest transitions in nineteenth century Austria and twenty-first century Lao PDR, we analyzed forest transitions as contested processes of shifting power relations, in which both the users and uses of forests experience a reduction in complexity, at the expense of multifunctional forest use and local forest users. We adapted the Theory of Access to develop a conceptual framework for analyzing the *practices* of multifunctional forest use and their benefits for local livelihoods as well as the *legal, structural and relational as well as social-metabolic mechanisms* that simplify and exclude these use(r)s in the course of both historical and contemporary forest transitions.

Our findings have important research and policy implications. Research on forest transitions, that tends to neglect issues of power, conflict and justice, can benefit from the analysis of power relations in future conceptual and empirical work to avoid outcomes that unidirectionally emphasize forest regrowth while neglecting the social costs and power shifts of such transitions. For global forest conservation and reforestation programs, our research shows that local livelihoods depend on forests in manifold, often unrecognized ways. In order to account for environmental justice conflicts, the integration and recognition of local land claims, but also alternative land use practices that integrate agricultural and forest lands are important to avoid exclusionary effects and develop more sustainable and just strategies for forest recovery.

## Footnotes

1 Although the separation of agricultural and forest land has indeed accompanied forest transitions since these were first observed for eighteenth century Europe, this process has received little attention in scholarly debate (for an exception, see, e.g., Bonan 2019).

2 We thus exclude “cultural” and “regulating” ecosystem services. Very likely, cultural ecosystem services are also more diverse in traditionally managed multifunctional forest landscapes (e.g., Gould et al. 2014) and thus reduced by excluding local people from forests. Some regulating ecosystem services, on the other hand, very likely improved in the context of forest transitions, even for local people, including, for example, the protection from natural hazards such as floods (Mather and Fairbairn 2000).

3 The different assessments are mainly caused due to differing definitions of forests. FAO data defines forests as land area with a tree canopy cover of more than 10% as opposed to 20% in national statistics in Lao PDR.

4 Whereas security concerns dominated resettlement in the 1970s and 1980s, the eradication of shifting cultivation and its increasing competition with commercial forestry and agriculture as well as conservation have dominated resettlement since the 1990s (Baird and Shoemaker 2007).

5 Although resettlement continued, the LFA policy emerged as the most important mechanism to impede shifting cultivation and turn rural people into more sedentary agricultural practices (Kenney-Lazar 2013, pp. 4–5).
